# Tailoring Strength and Ductility of a Cr-Containing High Carbon Steel by Cold-Working and Annealing

**DOI:** 10.3390/ma12244136

**Published:** 2019-12-10

**Authors:** Jing Wang, Yongfeng Shen, Yan Liu, Fuguo Wang, Nan Jia

**Affiliations:** 1 Key Laboratory for Anisotropy and Texture of Materials (Ministry of Education), School of Materials Science and Engineering, Northeastern University, Shenyang 110819, China; 2 The State Key Lab of Rolling & Automation, Northeastern University, Shenyang 110819, China; 3 Wuxi Tianchen Cold-drawn Steel Co. Ltd., Wuxi 214151, China

**Keywords:** high-carbon steel, strength, ductility, precipitate, cold drawn

## Abstract

SEM, TEM characterizations, in combination with tensile tests, provided an intriguing observation that ultra-high-strength and good ductility could be achieved simultaneously by changing the ratio of large and small precipitates in high-carbon steel (1.0C-1.5Cr-0.31Mn-0.20Si, wt %). The high yield strength of 670 MPa, tensile-stress of 740 MPa, and good ductility (elongation of 26%) were obtained by adopting spheroidization annealing, cold rolling, recrystallization annealing, and cold drawing. This led to nanosized precipitates with a large ratio of big size to the small size of 0.28, promoting high dislocation storage of 1.39 × 10^14^ m^−2^. In addition, the finite element (FE) method was used to simulate the cold-rolling process, and the largest stress and strain were 830 MPa and 0.6 at a depth of 3 mm after the fourth pass of the 0.10C-1.50Cr steel, respectively. The stress and strain accumulation in the top layer was potentially caused by severe plastic deformation, as well as attrition rendered by the rollers. This explained the emergence of dense low-angle grain boundaries in the region close to the surface of the cold rolled steel.

## 1. Introduction

Cold drawn high carbon steel wires have wide industrial applications, such as steel cords for automobile tires, cable wires for suspension bridges, ropes, and springs [1,2,3]. As a typical representative of hypereutectoid steel, 1.0C-1.5Cr steel has wide applications in high-speed steel and bearing steel because of its high strength [4,5]. High strength mainly results from the specific microstructure consisting of soft ferritic matrix and hard cementite lamellae. However, the lamellar pearlite is not appropriate for machining or cold forming. The spheroidization of cementite lamellae is beneficial to the cold formability of steels; hence, the pearlite steel is usually to be subjected to spheroidization annealing [4,6]. During annealing, the cementite lamellae gradually change their shapes into spherical particles to produce a mixed microstructure, in which globular cementite particles are uniformly distributed throughout the ferrite matrix [7,8,9].

In 1.0C-1.5Cr steel, each element has its irreplaceable role. Cr promotes the solid solution strengthening and reduces the spacing of pearlite lamellae, leading to work hardening [9]. Si induces the solid solution strengthening and suppresses the precipitation of proeutectoid network carbides, which are harmful to the strength and toughness of steel [10]. The rate at which silicon is rejected from the cementite phase limits the growth kinetics of pearlite. The characteristics of the two solutes have also been verified in the steels considered here. Al can effectively increase the eutectoid transformation temperature and the free energy change of transformation, preventing the initiation of crack and the formation of network carbide [10].

Several spheroidization annealing processes have been conducted to improve the work hardening ability of hypereutectoid steels, including isothermal spheroidization [6], intercritical spheroidization heat treatment [11], and cyclic heat treatments [12]. Many factors, such as the history of heat treatment [13,14,15], prior microstructure [16,17], chemical compositions [1,11,18], can influence the effect of spheroidization annealing. It was reported that the mean diameter of cementite particles could be reduced by suppressing the precipitation of grain boundary cementite and decreasing the pearlite interlamellar spacing of hot rolled steels [9]. Initial fine pearlite microstructure induces faster spheroidization, and the difference between coarse and fine pearlite microstructures arises from the higher defect density present in the latest [14]. On the other hand, the martensitic and bainitic microstructures spheroidize more rapidly than pearlite because the solute carbon distribution is more homogeneous in martensite and bainite, and the diffusion distances needed to form globular carbides are relatively short as compared to the diffusion distances in pearlite [16].

Generally, GCr15 bearing steel is subjected to cold deformation before heat treatment with the aim of shape-forming for bearings, such as cold rolling or cold forging. In fact, combining the prior cold deformation with phase transformation provides a novel approach for tailoring microstructures and improving the mechanical properties of high carbon steels. Lu et al. [19] combined the prior cold deformation with martensite pre-quenching and bainite transformation to obtain ultrafine martensite-bainite duplex microstructures in GCr15 bearing steel. The resultant impact toughness and fracture toughness were superior to that of the conventional quenching & tempering (QT) specimens. Chakraborty et al. [20] refined the thickness and size of bainitic sheaves and obtained significantly enhanced impact strength with high levels of tensile strength via the recrystallization of prior cold-deformed ferrite during austenitizing/austempering of SAE 52100 steel. Li et al. [21] showed that the prior cold deformation plus austenitizing treatment increased the impact of absorbed energy by approximately 7% in the spheroidized 1.0C-1.5Cr bearing steel. Recently, it was reported that Nb-Ti microalloying could be successfully used to suppress grain growth in gear steels for high temperature carburizing [22].

Since the combination of plastic deformation and phase transformation shows great potential to improve mechanical properties of bulk steels, in this study, spheroidization annealing, cold rolling, recrystallization annealing, cold drawing, and stress-relief annealing were conducted on 1.0C-1.5Cr bearing steel. We aimed to establish the relationship between mechanical properties and microstructures of the 1.0C-1.5Cr bearing steel and then drew an improvement map of the steel for industrial applications. In addition, a simulation based on Deform software was performed to investigate the evolution of stress and strain during the cold rolling process of the Cr-containing high carbon steel.

## 2. Materials and Methods

### 2.1. Sample Preparation

**Figure 1:**
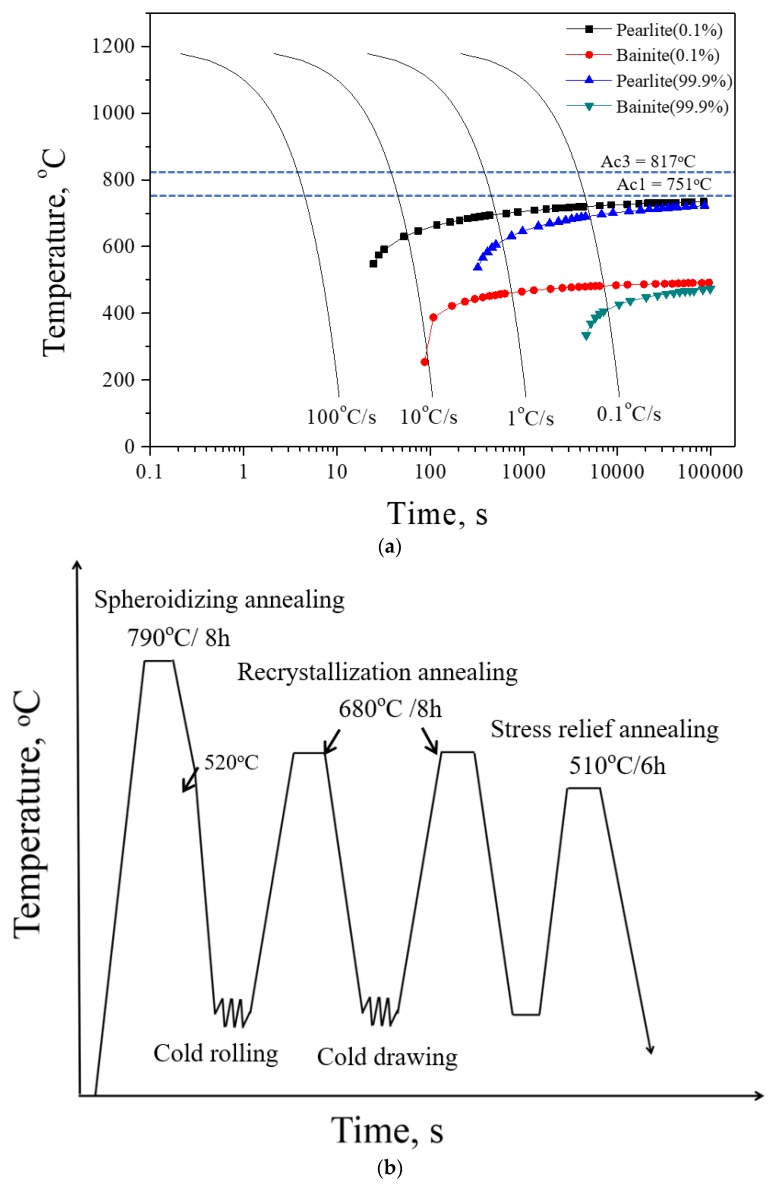
Continuous-cooling-transformation (CCT) diagram determined for the investigated steel with the nominal chemical bulk composition of 0.10C-1.50Cr-0.31Mn-0.20Si (wt %) (**a**); Schematic illustration of the different heat-treatment routes applied to the investigated steel (**b**).

Typical 1.0C-1.5Cr steel was used in this study. The alloy was melted in a vacuum induction furnace, and an ingot was cast (300 × Ф 50 mm^3^). The alloy’s chemical composition, measured by inductively coupled plasma mass spectroscopy, is listed in Table 1. The calculation using JMatPro software showed that the austenite-starting temperature (*A*_c1_) and austenite-finishing temperature (*A*_c3_) were 751 °C and 817 °C for the investigated steel. Using various cooling rates from 0.1 °C/s to 100 °C/s, the measured phase transformation diagram for the studied steel indicated (Figure 1a) that the bainite-start temperature (*B*_s_) and bainite-finish (*B*_f_) temperature were 320 °C and 480 °C, while those for pearlite were 520 °C and 730 °C at a cooling rate of 40 °C/s.

A 100 mm high bar was cut from the ingot, homogenized in an air furnace at 1150 °C for 1 h to remove the inhomogeneous microstructures, hot rolled at 950 °C in 4 passes to a diameter of 40 mm (*ε* = ~20%), and then cooled to room temperature in air. The bar was heated to 790 °C and kept for 8 h (spheroidized annealing) and cooled to 520 °C with the furnace, subsequently air-cooled to room temperature. The spheroidized annealed bar was cold rolled to a final thickness of 30 mm via 4 passes (*ε* = ~25%) and then heated to 680 °C for 8 to 10 h for recrystallization annealing treatment. The experimental steel after cold rolling and recrystallization annealing was cold drawn, then heated to 680 °C for 8~10 h for recrystallization annealing. At last, the steel was heated to 510 °C and kept for 6~8 h for stress-relief annealing (Figure 1b).

### 2.2. Microstructural Characterization

Metallographic observations were conducted by using a ZEISS ULTRA 55 field emission scanning electron microscope (FE-SEM); before observations, the samples were mechanically polished and then etched with 4% nitric acid. The morphology and the chemical composition of the microstructure were analyzed by scanning electron microscopy (SEM) mode using a JXA-8530F electron probe micro-analyzer (EPMA). The size of the carbide particles was measured using image-pro Plus and Photoshop according to the micrograph obtained by SEM. The two-dimensional cross-sectional area of each carbide particle was measured on a polishing plane, which was equivalent to a circular area, and the equivalent diameter of the particle was determined. At least 15 micrographs were taken with magnifications from 2000 to 6000 for each specimen. The total number of particles measured ranged from approximately 300 to 900, depending on the materials and process parameters. The particles larger than or equal to 700 nm were referred to as large-sized particles. The microstructure of 1.0C-1.5Cr steel after different treatments was analyzed by an electron backscatter diffraction (EBSD) system, and the EBSD data were post-processed by HKL CHANNEL 5 software. Samples for EBSD analysis were prepared by electrolytic polishing with a solution consisting of 10% perchloric acid and 90% ethanol.

Close observations of microstructural characteristics of 1.0C-1.5Cr steel after various processes were performed in a Tecnai G2 20 transmission electron microscope (TEM), operated at an accelerated voltage of 200 kV. The sample was cut into slices with a thickness of 500 μm and then mechanically thinned to 50 μm, subsequently electropolished by using twin-jet equipment in an electrolyte consisting of 10% perchloric acid and 90% ethanol at the temperature of −25 °C. It should be mentioned that the characterized regions were close to the surfaces of both cold rolled and cold drawn steels.

### 2.3. Mechanical Properties Tests

The dog-bone specimens were cut from the middle of as-annealed steels along the longitudinal direction using an electron discharge machine and mechanically polished using silica paper to the gage dimensions of 6 × 3 × 1.5 mm^3^. The uniaxial tensile test was performed to determine the mechanical properties of the samples under each processing condition at room temperature. The tests were carried out using an AG-X plus electronic universal testing machine at a strain rate of 3 × 10^−2^ s^−1^. Microhardness measurements were performed on a 401MVD hardness testing machine (Wolpert measuring instruments Ltd.) with a load of 300 g and a loading time of 20 s. The hardness value was averaged from 12 duplicated tests for each specimen.

### 2.4. Simulation of the Cold Rolling Process

Cold rolling process was simulated by using Deform Software (Scientific Forming Technologies Corporation, SFTC, Columbus, OH, USA), which is a simulation system based on finite element method (FEM) designed to analyze the complex metal forming process, microstructure and grain evolution, transient thermal response, residual stress, and distortion. It enables designers to analyze metal forming, heat treatment, machining, and mechanical joining processes on the computer. DEFORM has proven itself to be extremely effective in a wide range of research and industrial applications. Herein, we used the DEFORM system to predict the evolution of stress and strain vs. the cold rolling reduction, providing a visual stress/strain distribution map for guiding the practice. The initial parameters were as follows: the dimensions of 1.0C-1.5Cr steel were 40 × 40 × 40 mm^3^, the diameter of the working roll was 150 mm, and the rolling speed was 100 mm∙s^−1^. The rolling process was divided into 4 passes, and the reduction of 4 passes was 2.8, 2.4, 1.8, and 1.6 mm, respectively. The simulated plate was divided into hexahedral mesh division with 5300 meshes in total.

## 3. Results

### 3.1. Microstructure Characteristics

#### 3.1.1. Hot Rolled 1.0C-1.5Cr Steel

**Figure 2:**
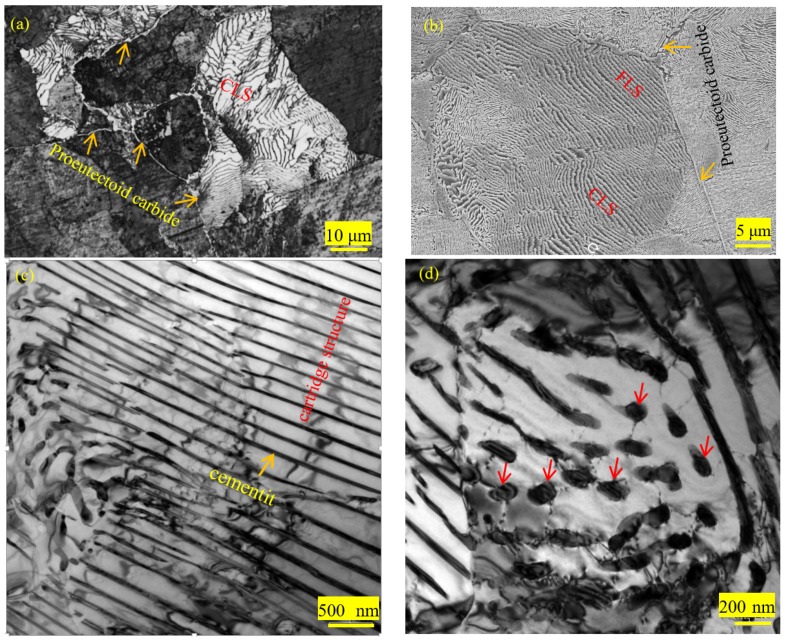

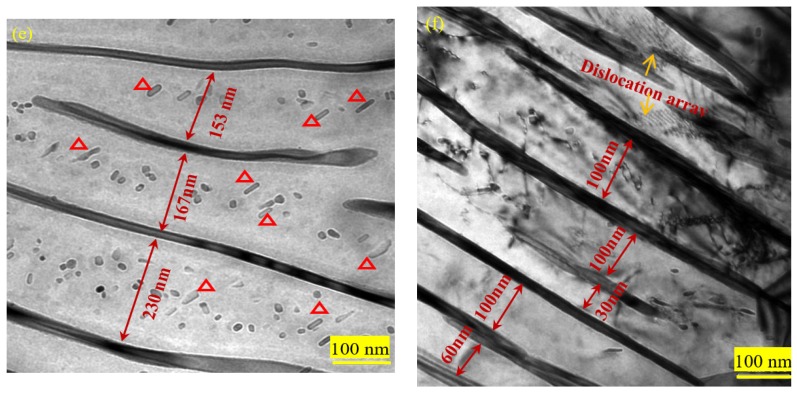
(**a**) Optical pictures, (**b**) SEM observation, and (**c**–**f**) TEM images.

Figure 2 shows the microstructural features of the hot rolled 1.0C-1.5Cr steel. One could clearly see that netlike pearlite and pre-eutectoid carbide coexisted in the as-hot rolled steel. Nevertheless, pre-eutectoid carbide mainly located at grain boundaries, leading to grain boundaries light (arrows indicated in Figure 2a,b). During cooling, the carbide network first precipitated along grain boundaries of the austenite, and then the carbide network gathered to form a banded structure during the subsequent rolling. It was reported that the pro-eutectoid carbides were dendritic, and the dendrite arms preferentially grew along the grain boundary interface [23]. Two different types of structures appeared in pearlite, namely coarse lamellar structure (CLS) and fine lamellar structure (FLS) (Figure 2a,b). The different stress states during the rolling process should be responsible for the appearance of these distinct morphologies. The cementite lamella underwent bending deformation during the rolling process as the pearlite lamellar was at a large angle with the rolling direction. In contrast, pearlite remarkably refined to form FLS as the pearlite lamellae were at a small angle or parallel to the rolling direction [24].

TEM observations were performed to characterize the microstructures of the hot rolled 1.0C-1.5Cr steel in detail, as shown in Figure 2c–f. The ferrite-cementite interface in pearlite was not completely straight, revealing a curvature difference in the local area. Partially adjacent ferrite lamellae were interrupted by fine cementite lamellae in localized regions, forming a cartridge-like structure (Figure 2c). Wang et al. [25] used computer-aided three-dimensional (3-D) tomography to observe the three-dimensional morphology of pearlite, showing that the pearlite lamellar structure was not perfect, with holes in the cementite sheet. These holes were high curvature layered fault regions with high chemical potentials in which cementite preferred to dissolve and nucleate, resulting in irregular surface of the cementite sheet [26]. In addition, intermittently rod-shaped or spherical cementite (arrows indicated) was observed in the localized region (Figure 2d), which was related to the degeneration of pearlite because the pearlite was difficult to retain the way of collaborative growth at low transformation temperature [27,28]. Cementite exhibited as spherical and rod-like when the diffusion rate of alloying elements was slow because of low temperature. Zhang et al. suggested that the presence of branches in a localized area of a single cementite sheet was caused by branching growth [28]. Provided larger thickness of cementite lamellae, more carbide forming elements were consumed during the process of co-grow in coordinate with the adjacent ferrite sheets. Thus, a branch appeared in the local area of a single thicker cementite sheet to maintain the synergistic growth of pearlite, leading to the formation of two thinner cementite sheets [28].

Interestingly, close TEM observations showed that a large number of cementite particles with tens of nanometers in spherical interspersed among the lamellar cementite (Figure 2e,f). The thicknesses of lamellae varied from 10 nm to 20 nm. Dislocations arrays could be seen in a few ferrite plates, caused by the blockage of thin cementite lamella to dislocation movement (indicated by arrows in Figure 2f). As a soft phase, plastic deformation initially occurred in the ferrite associated with dislocation multiplication and slip. Dislocations moved freely in the ferrite zone and aggregated along the interface between ferrite and cementite with increasing strain [29]. However, it was difficult to cross-slip in cementite and thus accumulated at the tip of cementite or cut through it, leading to broken cementite lamellae [10]. On the other hand, at high temperature, austenite could introduce a large number of dislocations in the ferrite phase of pearlite during hot rolling. These dislocations were effective paths for element diffusion and cementite nucleation. Maintaining a straight interface of cementite must increase the system’s free energy. Cementite tended to break into spherical or short rod-like dispersed in the ferrite for reducing the interface energy, leading to the thickening of ferrite lamellae [30]. It could be seen that the thicknesses of ferrite lamellae varied from 30 nm to 230 nm (Figure 2e,f). It was suggested that the precipitation of cementite particles at dislocations had a higher driving force, resulting in the disappearance of the original dislocations [30]. This is the reason why some ferrite lamellae had dislocations, and others were free of dislocations. Besides, the coarsening of the cementite lamellae could be carried out by using dislocations to consume the adjacent cementite lamellae. These dislocations accelerated the degradation of pearlite and even accelerated the spheroidization of the cementite at the initial stage of spheroidization annealing [30].

#### 3.1.2. Cementite Size in 1.0C-1.5Cr Steel after Different Processes 

**Figure 3:**
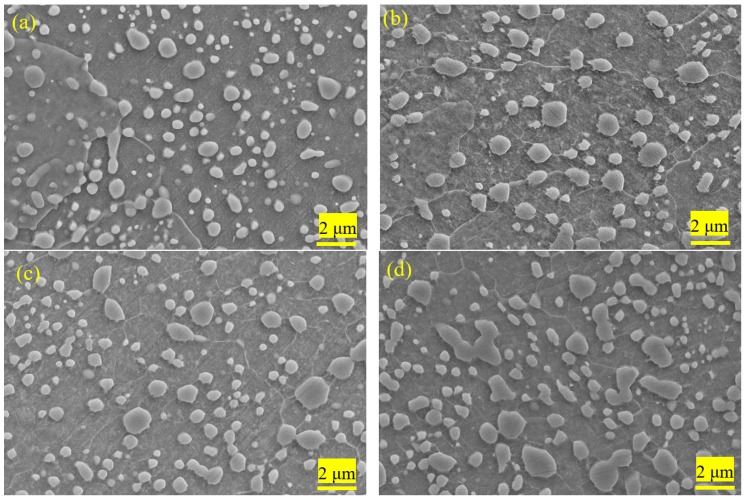

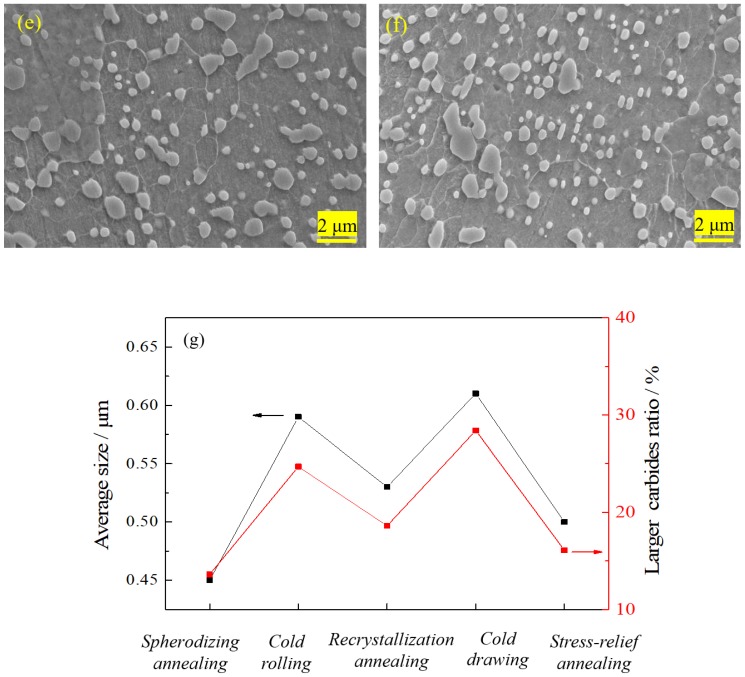
SEM images showing the morphology and the distribution of spheroidized cementite in 0.10C-1.50Cr steel after different processes: Spherodizing annealing (**a**), cold rolling (**b**), annealing (**c**), cold drawing (**d**), recrystallization annealing (**e**), and stress-relief annealing (**f**). Statistical results reveal the size and ratio of larger particles (**g**).

Figure 3 shows the average size of the spherical cementite and the ratio of large particles in 1.0C-1.5Cr steel after different processes. The ratio of the large particle was defined as the ratio of the particles with a size larger than ≥700 nm to total particles. One could see that the morphology and size of particles varied with different processes, especially the size distribution (Figure 3a–f). Obviously, the microstructure was mainly composed of ferrite matrix and undissolved spheroidized cementite. The dissolution of cementite was controlled by the diffusion of carbon and chromium [31]. The partially undissolved particles exhibited as elongated and irregular shapes because spherodizing annealing was conducted at the intercritical zone (i.e., between Ac1 and Ac3), leading to the incomplete dissolution and unevenly distributed of cementite. The smaller multi-pass cold deformation led to the coalescence of adjacent carbides, showing an irregular shape. It could be seen that the amount of 100~300 nm cementite particles decreased after spheroidization annealing, and the size of undissolved spheroidized cementite particles was mostly located in the range of 300~500 nm. Statistical results showed that the average size of cementite after spherodizing annealing was the smallest at 450 nm, with the smallest ratio of large particles at 12%. In contrast, the average sizes of cementite after cold rolling and cold drawing were large at 630 nm and 600 nm because of the elongation along the rolling/drawing direction. The ratio of large particles was 25% and 28% after cold rolling and cold drawing, respectively. It is worthy to point out that the real size was smaller than the statistical size because of the limited resolution of SEM. On the other hand, recrystallization annealing and stress-relief annealing slightly reduced the size non-uniformity and the ratio of larger particles.

#### 3.1.3. EBSD Analysis of 1.0C-1.5Cr Steel after Different Processes

**Figure 4:**
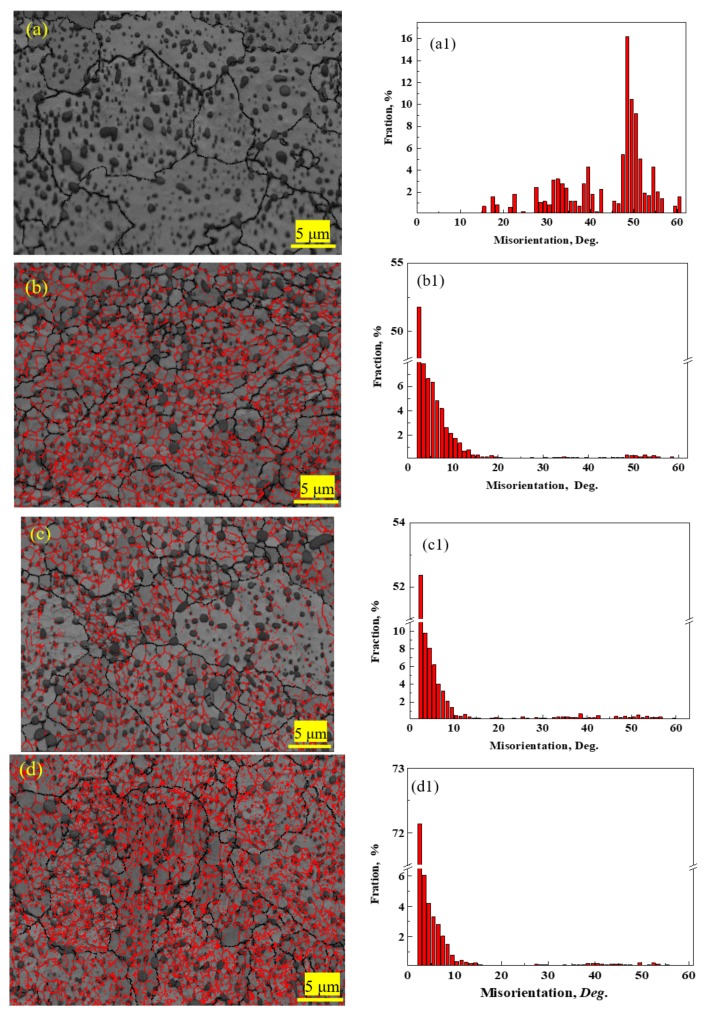
Image quality maps and misorientation angle distributions of 0.10C-1.50Cr steels at different stages: (**a**,**a1**) spherodizing annealed, (**b**,**b1**) cold rolled, (**c**,**c1**) recrystallization annealed, and (**d**,**d1**) cold drawn. Black lines indicate high-angle grain boundaries (HAGBs, ≥10°), and red lines for low-angle grain boundaries (LAGBs, <10°).

The microstructures of 1.0C-1.5Cr steel after various processes were studied using EBSD, as shown in Figure 4. After spheroidization annealing, few low-angle grain boundaries (LAGBs, <10°) were observed (Figure 4a,a1). However, there were a large number of LAGBs in the cold rolled 1.0C-1.5Cr steels, and the fraction of LAGBs was as high as ~80% (Figure 4b,b1). Recrystallization annealing only eliminated part of LAGBs, with a fraction of 70% (Figure 4c,c1). Interestingly, the fraction of LAGBs after cold drawing significantly increased to ~90% (Figure 4d,d1). Besides, the image quality map clearly showed that a few undissolved carbides existed at the boundaries of ferrite, connected with LAGBs (Figure 4b–d). The observed phenomenon must be related to the difference between boundary diffusion and volume diffusion of elements that might occur during cold deformation. This is discussed in Section 4.1.

#### 3.1.4. EPMA Maps and Energy Dispersive Spectroscopy (EDS) of 1.0C-1.5Cr Steel after Cold-Working

**Figure 5:**
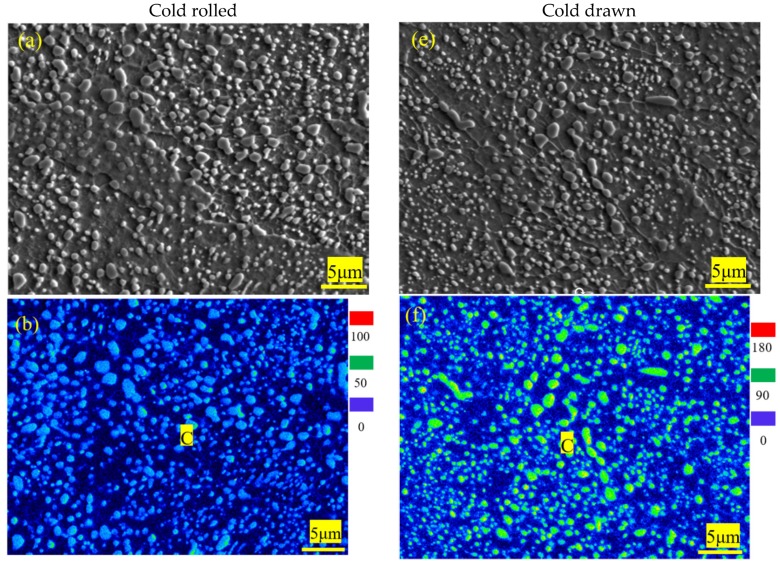

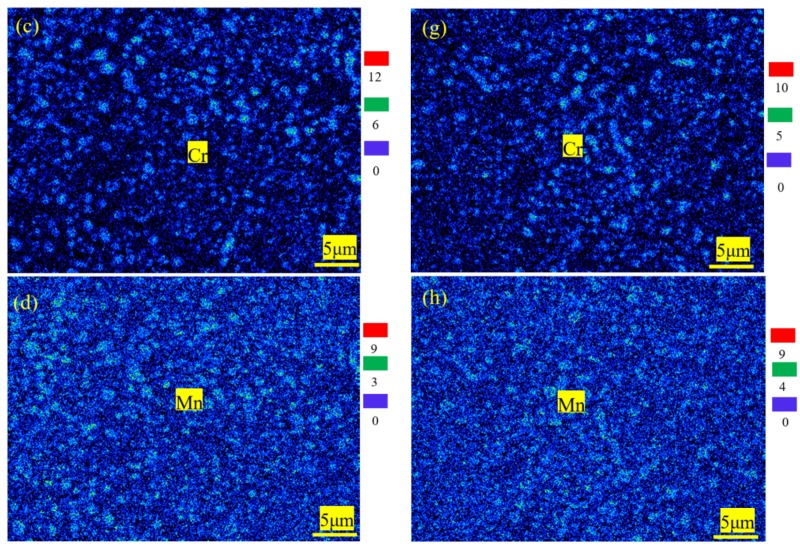
SEM images and electro-probe microanalysis (EPMA) maps of C, Cr, Mn distribution in 0.10C–1.50Cr steels after cold rolling (**a**–**d**) and cold drawing (**e**–**h**).

**Figure 6:**
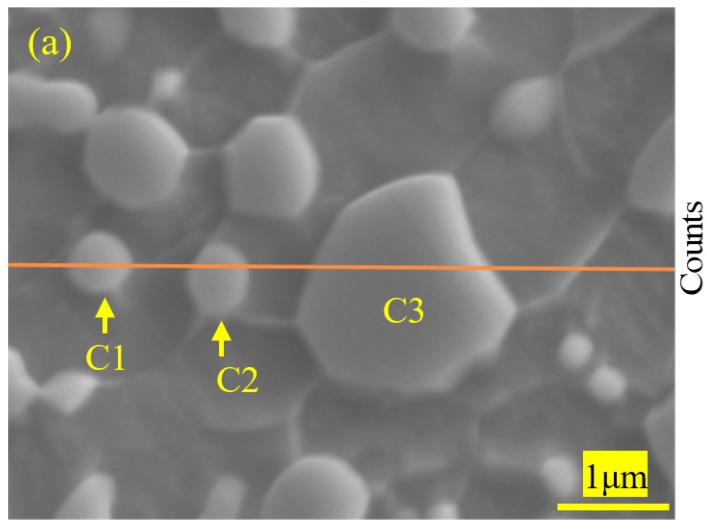

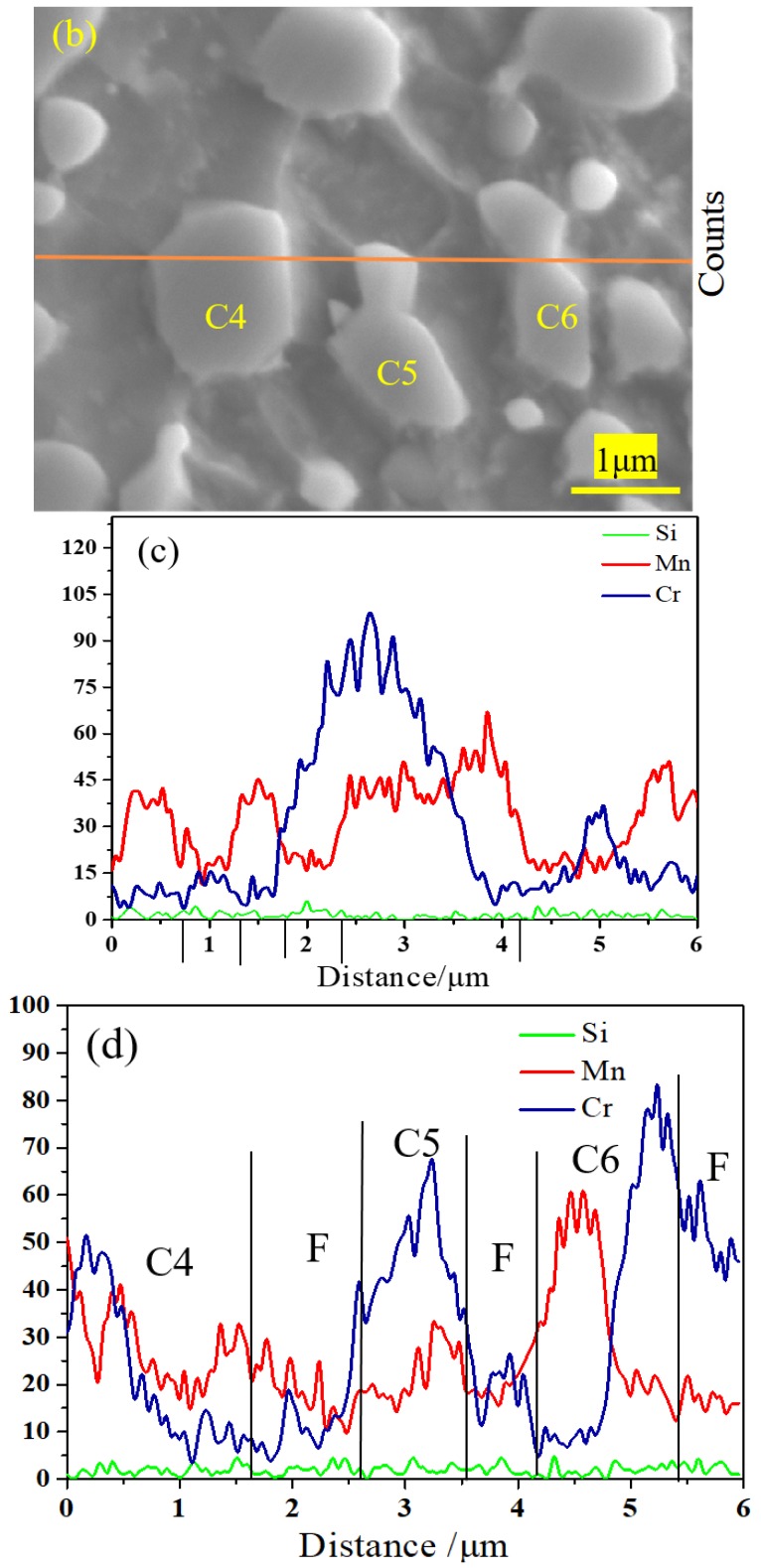
SEM image with the trace of the line scan and energy dispersive X-ray (EDX) analysis of cementite in 0.10C-1.50Cr steel: (**a**,**b**) cold rolling, (**c**,**d**) cold drawing.

Figure 5 shows SEM images and EPMAmaps of C, Cr, Mn distributions in 0.10C-1.50Cr steels after cold rolling and cold drawing. The distributions of elements were obviously heterogeneous. Because Cr is a medium carbide forming element and Mn has high diffusivity and solubility in cementite, spheroidized carbides mainly consisted of (Fe, Cr) _3_C, which was richened in Cr, Fe, and C (Figure 5). The size of the manganese atom is smaller than that of chromium atom, so the diffusivity of the chromium atom is smaller than that of manganese atom at a lower temperature. Thus, the manganese content in cementite was generally higher than that in the surrounding ferrite phase. Compared to cold rolling, the deformation degree of cold drawing was larger and faster, and the effect of thermal stress was obvious (Figure 5). Figure 6 exhibits SEM image with the trace of the line scan and energy dispersive X-ray (EDX) analysis of cementite in 0.10C-1.50Cr steels after cold rolling (a, b) and cold drawing (c, d). Six carbides were analyzed, which were labeled as C1, C2, and C3 in the cold rolled 0.10C-1.50Cr steel, while those in the cold drawn specimen were labeled as C4, C5, and C6. Because of the low solubility of carbon in ferrite, the interface between cementite and ferrite could be clearly distinguished by the distribution of carbon atoms. In addition, carbon enrichment in ferrite grain boundaries was evident, confirming that grain boundaries were important diffusion channels in the process of dissolution and coarsening of partial cementite caused by cold deformation (Figure 6a,c). The EDX line scanning illustrated that Mn content in large cementite was significantly higher than in small particles of cementite, and Cr content gradually increased from small to large cementite. Si content in cementite was lower than in surrounding ferrite, as shown in Figure 6b,d. This suggested that carbide forming elements Mn and Cr in ferrite diffused into cementite, while Si in cementite diffused into ferrite. Therefore, the partition of the alloying element retarded the growth of cementite dynamically.

#### 3.1.5. TEM Observations

**Figure 7:**
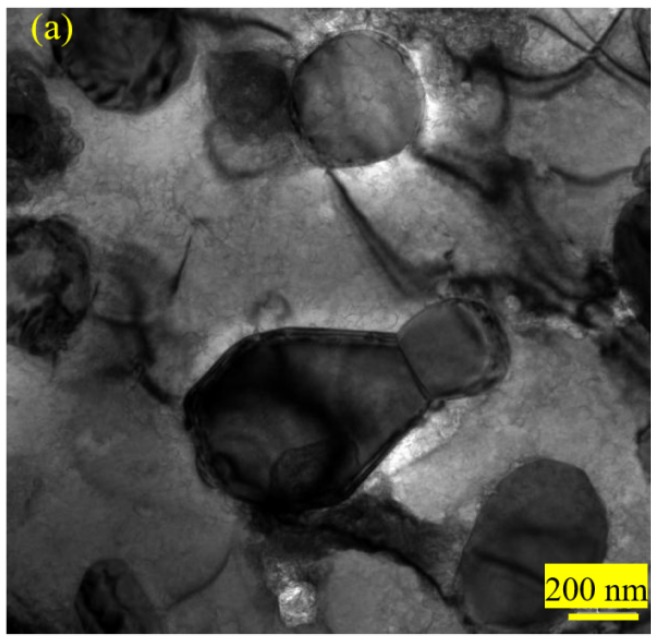

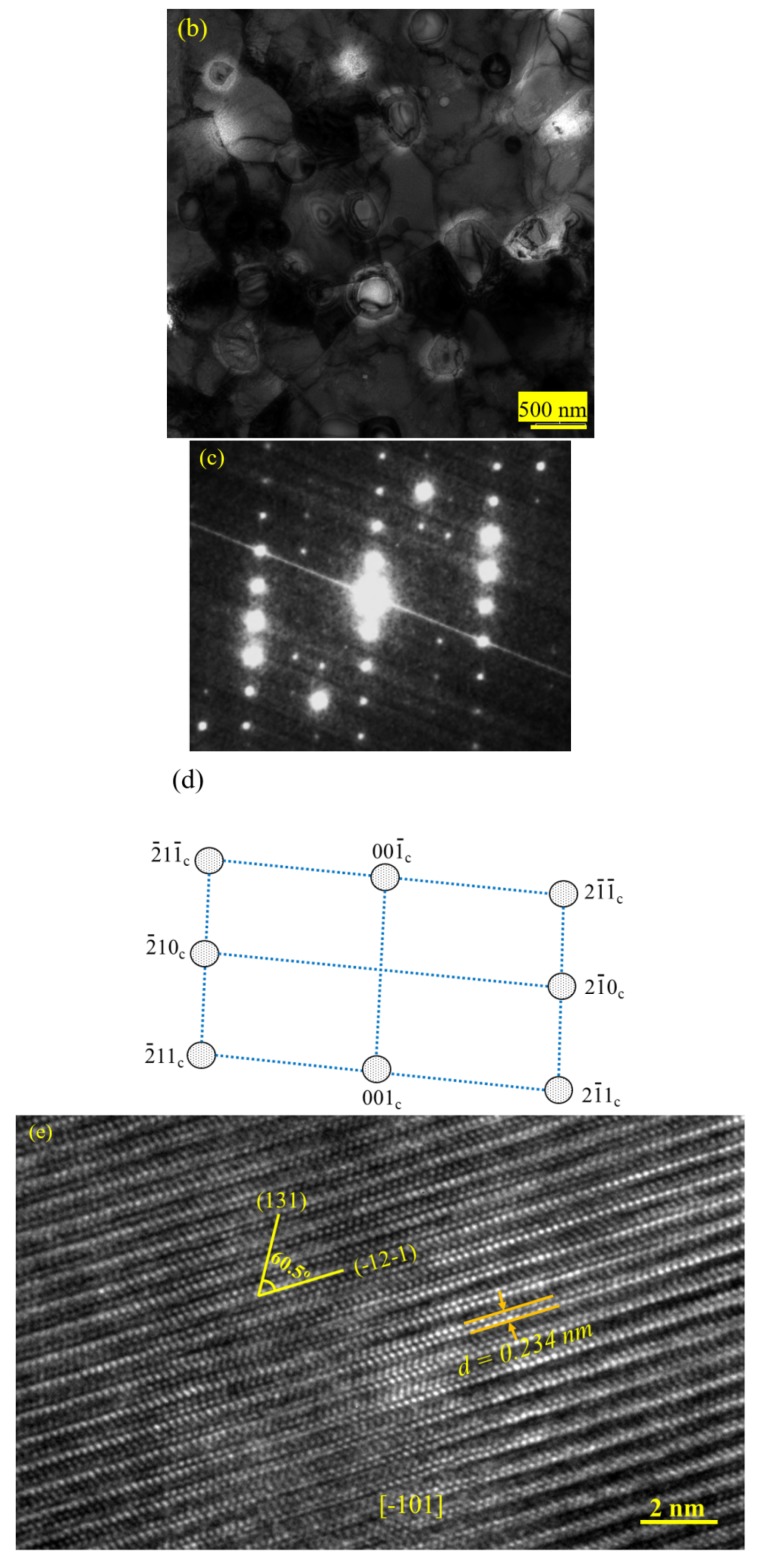
TEM micrographs show the morphology of 0.10C-1.50Cr steels after spheroidizing annealing (**a**), and recrystallization annealing (**b**). A selected area electron diffraction (SAED) pattern (**c**), a schematic illustration of the indexed SAED pattern (**d**), and high resolution image of cementite particle (**e**).

**Figure 8:**
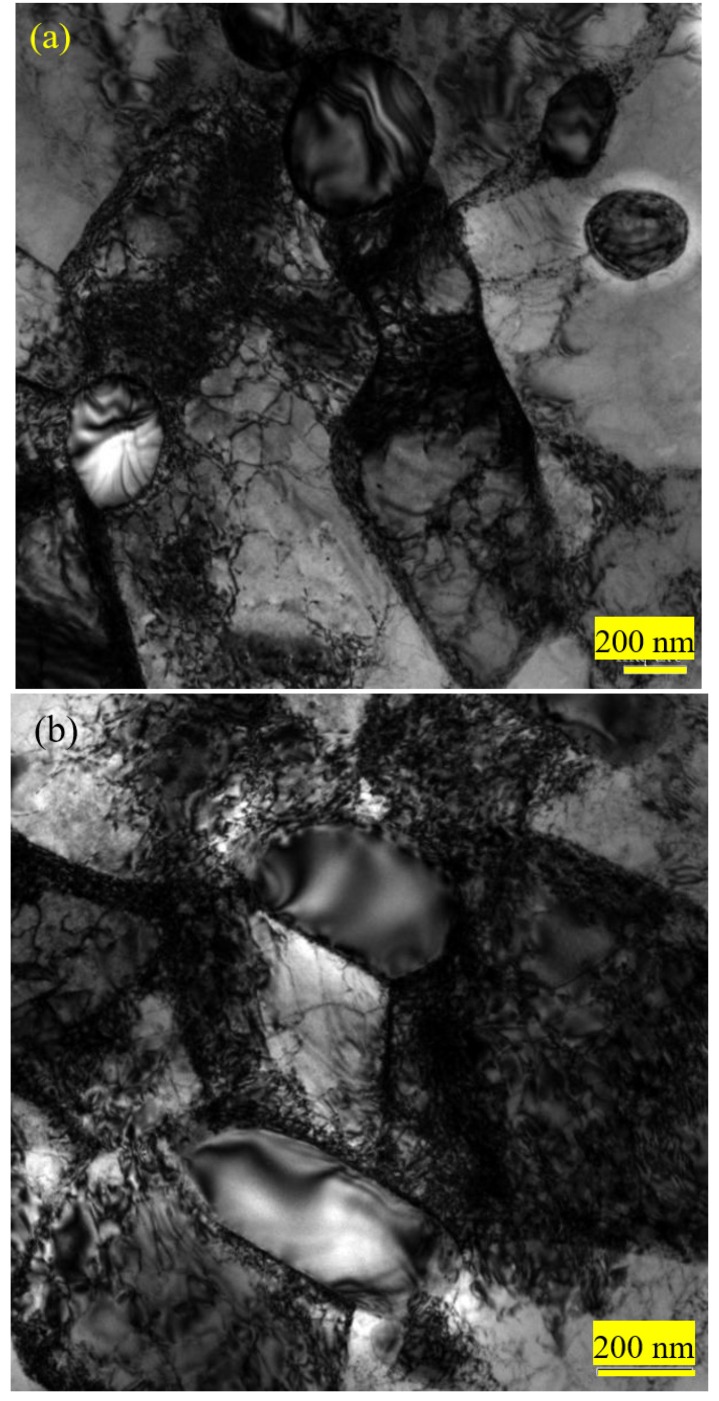
TEM micrographs show the morphology of 0.10C–1.50Cr steels after cold rolling (**a**) and cold drawing (**b**).

TEM micrographs showed that the microstructures of 0.10C-1.50Cr steel after spherodizing annealing consisted of ferrite matrix and spherical or irregular cementite in size of a few hundred nanometers. The surrounding of the cementite particles was clean (Figure 7a). After recrystallization annealing, the small particles were in the majority, and the size distributions of cementite became homogeneous. Interestingly, numerous particles emerged with circles, indicating that a dissolution process of cementite occurred during recrystallization annealing (Figure 7b). The dissolution of smaller cementite is beneficial to decrease the total interfacial energy per unit volume [32]. Figure 7c exhibits the selected area electron diffraction (SAED) taken along (120) zonal axis of cementite (i.e.,(111) zonal axis of ferrite), and Figure 7d presents the marked patterns. Obviously, the Pitsch-Petch orientation relationship was fulfilled between the cementite and the ferrite because the diffraction pattern of Fe_3_C particles overlapped with the surrounding ferrite matrix [33,34]. A high-resolution TEM image of the cementite particle revealed that the streaks were the typical characteristic of Fe_3_C with an interplanar spacing of 0.234 nm at {001}_Fe3C_, as shown in Figure 7d. The bulge of atoms array must be related to the difference of carbon content. Figure 8 exhibits the morphology of 0.10C-1.50Cr steel after cold rolling and cold drawing, respectively. There was a significantly high dislocation density in the cold drawn steel than in the cold rolled steel. The key reason was that there was a large level of deformation during cold drawing. The isotropic hardening stress component is proportional to the square root of the statistical stored dislocation density (*ρ*) [34]:

(1) $$\sigma_{f}=MaGb\sqrt{\rho }$$

here *α* is a factor related to the dislocation structure with a mean value of 1, *M* is the average Taylor factor of 3.06, *G* is the shear modulus of 72 GPa for ferrite steel, and *b* is the Burgers vector of 2.85 × 10^−10^ m. The estimated dislocation density was 1.39 × 10^14^ m^−2^ and 1.08 × 10^14^ m^−2^ for the cold drawn steel and the cold rolled steel, respectively.

### 3.2. Mechanical Properties

**Figure 9:**
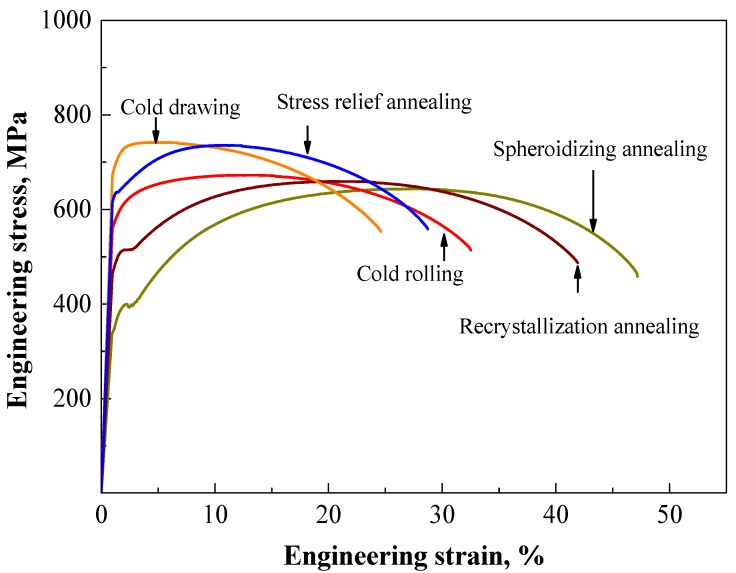
Engineering stress-strain curves of the experimental steel after different processes.

Engineering stress-strain curves for 0.10C-1.50Cr steels after different processes are plotted in Figure 9. Obvious yield points occurred in the stress-strain curve of 0.10C-1.50Cr steel after spheroidizing annealing, which could be attributed to dislocation accumulation on ferrite grain boundaries and dislocation activation in ferrite grains. The strength decreased, and the elongation increased after spheroidization annealing. This was mainly attributed to the spheroidization of lamellar cementite and the elimination of grain boundary network cementite. For the cold drawn steel, the tensile yield strength *σ_y_* (at 0.2% offset) was high at 670 MPa, and the tensile strength (*σ_TS_*) was 740 MPa, while the steel after stress-relief annealing had *σ_y_* of 600 MPa and *σ_TS_* of 740 MPa. In contrast, the spheroidizing annealed steel had a relatively low *σ_y_* of 340 MPa and *σ_TS_* of 640 MPa, while the steel after recrystallization annealing had a medium *σ_y_* of 470 MPa and *σ_TS_* of 660 MPa. It is interesting to note that the elongation-to-failure (*ε_f_*) increased considerably with decreasing *σ_y_*. For example, for the cold drawn steel with the highest *σ*_y_ at 670 MPa, the *ε_f_* value was only 26%. However, for the cold rolled steel with a low *σ_y_* of 565 MPa, the *ε_f_* value significantly increased to 37% because of a decrease in dislocation density. In contrast, for the spheroidizing annealed steel with a low *σ_y_* of 340 MPa, the highest *ε_f_* of 48% was related to the reduction in the amount and size of large particles after spheroidizing annealing (Figure 3g).

Tensile results for 0.10C-1.50Cr steels with different processing are summarized in Table 2. Microhardness (*H*_v_) measurement results agreed reasonably well with the tensile data. In summary, the strength decreased, and the elongation increased after spheroidization annealing. This was mainly because of the spheroidization of lamellar cementite and the elimination of grain boundary network cementite. After cold rolling, the strength of 0.10C-1.50Cr steel increased, and the elongation decreased. The plasticity after cold drawing decreased because the average size and the ratio of larger cementite particles were larger than those after cold rolling (Figure 3g), and the increasing strength was associated with the high dislocation density in the cold drawn steel (Figure 8b). Generally, cold deformation leads to work hardening, introducing a large number of low-angle grain boundaries, and some cementite particles are dissolved, coarsened, and combined. After the heat treatment, the size of cementite particles decreased and became uniform, but the ferrite grains became coarser.

### 3.3. Simulation of Cold Rolling Process

The finite element (FE) method based on DEFORM-3D was used to simulate the cold-rolling process, and a thermo-coupled viscoplasticity model was used to calculate the stress and strain in cold rolling. The fundament mechanism is assumed that stress is a function of strain during cold rolling [35]:

(2) $$\sigma =\left\{ \begin{array}{l} 219.1\times 10^{3}\varepsilon ,\;\varepsilon \leq 0.001856\\ 847\varepsilon^{0.129}+30.37,\;\varepsilon >0.001856 \end{array}\right.$$

**Figure 10:**
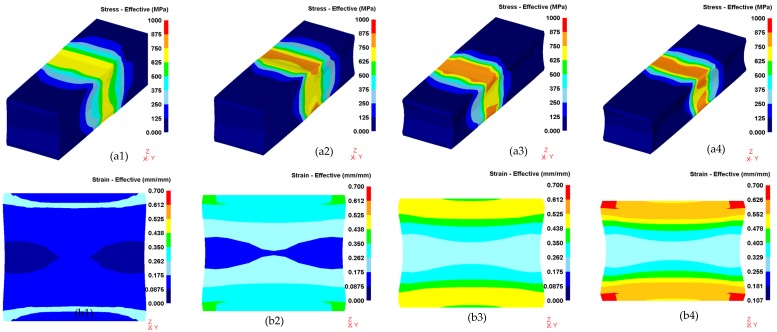
Finite element (FE) simulations of the cold-rolling process for the 0.10C-1.50Cr steel. Cross-sectional views show the distributions of equivalent stress (**a1**–**a4**) and strain (**b1**–**b4**) during cold rolling: (**a1**,**b1**) the first pass, (**a2**,**b2**) the second pass, (**a3**,**b3**) the third pass, and (**a4**,**b4**) the fourth pass.

**Figure 11:**
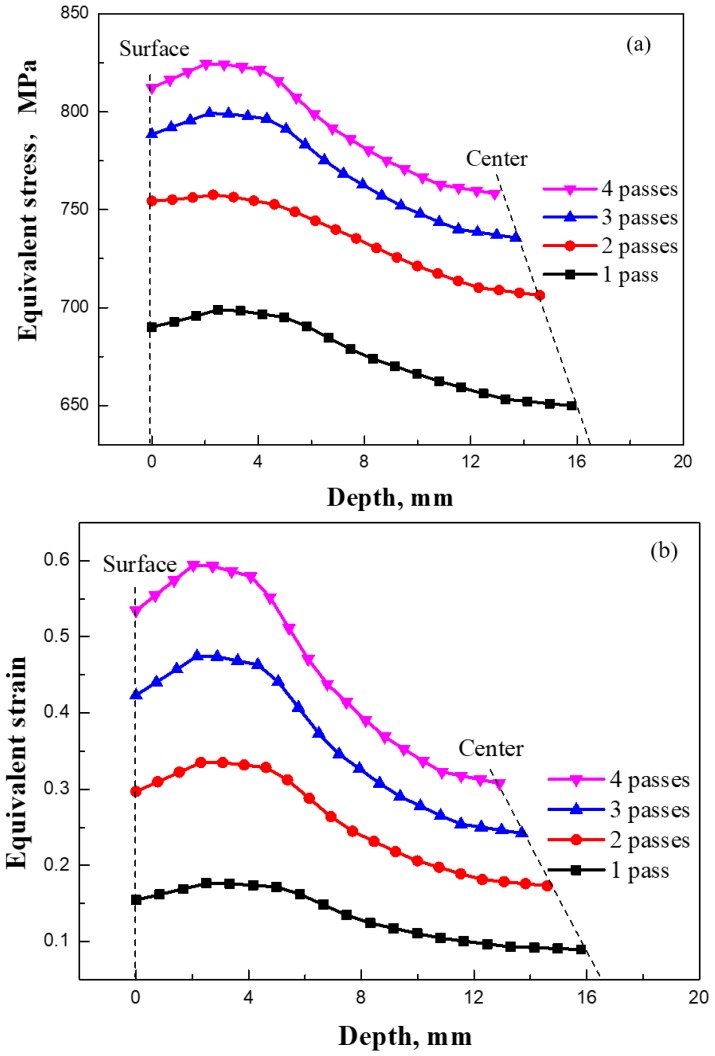
Equivalent stress (**a**) and strain (**b**) at different cold rolling passes as a function of depth from the surface to center of the 0.10C-1.50Cr steel.

Parameters in the calculation include: density *ρ* = 7.6 kg·m^−3^, elastic modulus *M* = 142 GPa, and Poisson ratio *ν* = 0.33. Figure 10 shows the distributions of equivalent stress (a1–a4) and strain (b1–b4) during cold rolling of the 0.10C-1.50Cr steel. It could be seen from the cross-sectional view that the equivalent stress and strain significantly increased with increasing passes. Interestingly, the equivalent strain was the largest at the corner of the plate after the fourth pass (Figure 10b4). The corresponding values are summarized in Figure 11. First, the stress and strain increased with increasing passes. Second, at the beginning of each pass, the stress and strain increased with increasing depth from the surface to a depth of ~3 mm, and subsequently, the values decreased with increased depth. The largest stress and strain were 830 MPa and 0.6 at a depth of 3 mm after the fourth pass of the 0.10C-1.50Cr steel. The accumulated stress and strain in the top layer were potentially caused by severe plastic deformation, as well as attrition rendered by the rollers, leading to the occurrence of dense LAGBs on the top layer.

## 4. Discussion

### 4.1. Cementite Particles Size

It is well known that the boundary diffusion of elements is much faster than the volume diffusion [36]. Cold deformation can produce seriously distorted ferrite grains containing either dense dislocations or dislocation cells [37]. Cold deformation generates a large amount of thermal stress, which raises the temperature of the sample. Hence, the presence of these LAGBs is beneficial to the diffusion of elements, such as carbon and chromium, accelerating the coarsening of cementite [37]. Several research groups [38,39,40] reported that cementite has a tendency to precipitate and coarsen along the boundaries of pre-austenite, martensite, and sub-grain. Thus, the dissolution and coarsening of cementite occur simultaneously during cold deformation. In the current case, LAGBs with a high density had already been introduced into the region close to the surface of the cold rolled plate due to the inhomogeneous strain distribution throughout the normal direction of the plate (see the FE simulation results). With the propagation of deformation/strain, small cementite particles dissolved, while the larger ones became even larger, leaving the undissolved cementite at the ferritic LAGBs. The substantive characteristics of the coarsening process of cementite showed that large particles swallowed up small ones. Moreover, the coarsening process was associated with a decrease in interface energy between cementite and matrix, as the dissolution of cementite particles was useful to decrease the interface area per unit volume. However, carbon concentration in the matrix increased with the dissolution of cementite and reached a saturation state in the end [21]. In order to reduce the interfacial energy, the remaining cementite particles were coarsened, and the coarsening process of cementite particles during cold deformation was similar to that of Ostwald ripening [32]. In general, the coarsening of cementite was promoted by a long holding duration at high temperatures. Obviously, cold working accelerated this process so that it occurred at lower temperatures and short durations, as demonstrated in Section 3.1.2, Section 3.1.4, and Section 3.1.5.

### 4.2. Microstructure-Mechanical Properties Relationship

As above mentioned, ferrite and brittle twisted cementite lamellae were alternately arranged (Figure 2). During cold deformation, soft ferrite deformed in priority, but the limited deformation of cementite restricted deformation of the ferritic matrix, leading to an inhomogeneous distribution of strain in the lamellar pearlite. Moreover, a hard and brittle network of the pre-eutectoid cementite precipitated at grain boundaries, splitting the continuity of the matrix. Thus, an obvious cleavage surface formed during deformation [41]. Subsequently, cracks initiated at the network of brittle cementite, which aggravated the failure of the brittle cleavage. Hence, the plasticity of the lamellar pearlite structure was poor, and the stress-strain curve showed an insignificant yield point accordingly (Figure 9). In contrast, the stress-strain curve of the specimen (with lamellar pearlite structure) after spheroidizing annealing showed obvious yield points, which was attributed to dislocation accumulation on the ferritic grain boundaries and dislocation activation in ferrite according to the polycrystalline yield theory [42]. After heat treatment, the strength of the steel decreased, and the elongation increased. The fundamental reason must be related to the dispersed cementite particles in the fine-grained ferritic steels. In contrast, the strength of the 0.10C-1.50Cr steel increased, and the elongation decreased after cold rolling and cold drawing. The decreasing strain after the cold drawing was due to the fact that the average size and the ratio of larger cementite particles were larger than that after cold rolling (Figure 3g). On the other hand, the increasing strength was associated with the high dislocation density in the cold drawn steel (Figure 8b).

Mechanical properties of pearlitic steels were mainly affected by its microstructure, including the size of pearlite colonies and the thickness and spacing of cementite lamellae [43]. However, these microstructural parameters were dominated by the chemical composition of the steel and the history of working and heat treatments [36]. The different mechanical properties were because of different sizes of ferritic grains and cementite particles in each steel. As far as the spheroidized steel is concerned, mechanical properties are determined by the sizes of ferrite grain and cementite particles, as well as the misorientation angle of ferritic grain boundaries and the distribution of cementite particles [44]. Cold deformation led to work hardening by introducing a large number of low-angle grain boundaries. Meanwhile, a few cementite particles dissolved, coarsened, and combined during cold working. After heat treatment, the size of cementite particles decreased and became uniform, but the ferritic grains became coarser. The microstructures consisted of spherical submicron-sized cementite, which was dispersedly distributed in the ferritic matrix. This led to a good match between strength and plasticity [41].

**Figure 12:**
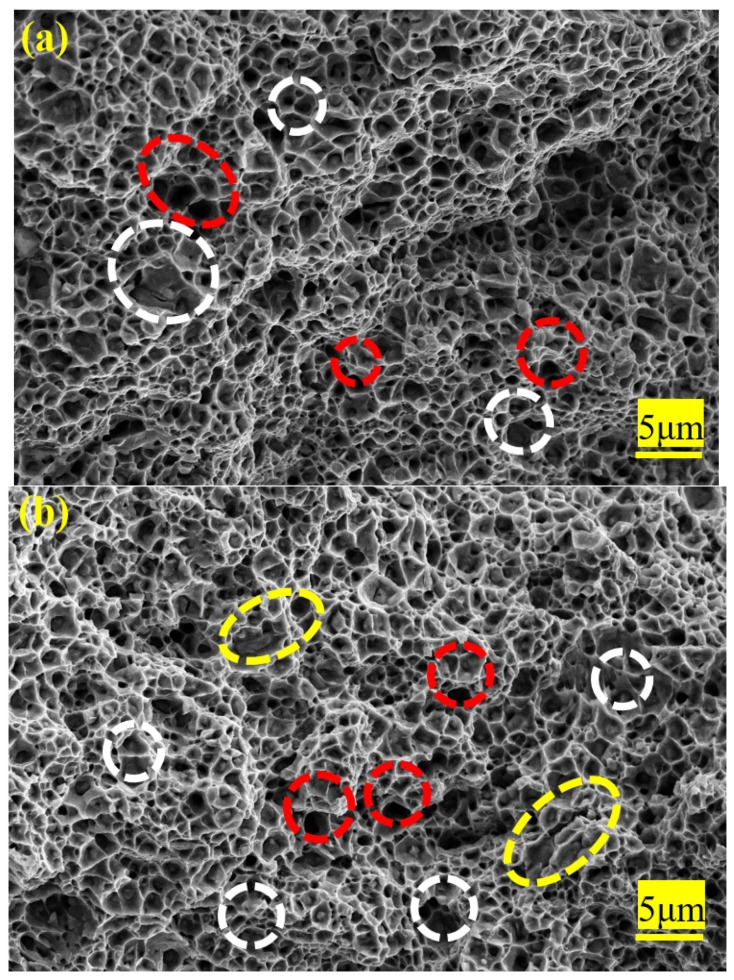
Fracture surface morphology of the tensile samples: (**a**) cold rolling, (**b**) cold drawing. Red circle: Microvoids; white circle: Cementite in dimples; yellow circle: Irregular thin plate-like particles.

SEM images of the fracture surface of the cold rolled and cold drawn specimens after tensile tests are shown in Figure 12a,b, respectively. One could see that there were numerous undissolved cementite particles and dimples in the fractured region. Undissolved cementite particles were found in a few dimples. It could be deduced that the larger number and the smaller size of dimples were responsible for the larger elongation of the 33.6% cold rolled specimen and the 25.8% cold drawn specimen. During tension, the fine particles (spherical or nearly spherical) promoted the nucleation of dimples through microvoid coalescence. The presence of dimples supported that a microporous-aggregation-related failure mode occurred during tensile tests. In contrast, strip-like carbides were against toughness compared to spherical ones because the strip-like carbides located at ferritic grain boundaries might reduce the boundary cohesion [45,46]. When the material was subjected to external stress, the dislocations accumulated around strip-like carbides, resulting in stress concentration. However, irregular thin plate-like particles generally had a larger contact area and a higher volume ratio with the matrix, while dislocation movement via cutting cementite particles inevitably generated new surfaces, leading to the increasing system energy and thus the more significant strengthening effect [41]. This was the main reason why the strength of the specimen undergone cold drawing was higher than that undergone cold rolling.

During the tensile test, microcracks nucleated at the interfaces between the coarse cementite and the ferritic matrix. With the propagation of deformation, the microcracks aggregated to form microvoids. A few microvoids were related to the fallen off of undissolved cementite particles. Furthermore, the dissolution of partial cementite particles during cold deformation increased carbon content in the ferritic matrix and thus increased the strength of ferrite. As a result, the interfacial strength of some ferrite-cementite was lower than that of the ferritic matrix. The coarsening of cementite particles increased the stress concentration at the interface between the ferritic matrix and cementite during deformation, resulting in an increasing tendency of microcrack nucleation. It has been shown that during the cold rolling of the spheroidized bearing steel, microcracks nucleate at the interfaces between the larger cementite particles and the ferritic matrix [1].

## 5. Conclusions

The motivation of this study was to obtain the best combination of strength and ductility by controlling the ratio of small and large cementite particles in the microstructure. The study indicated that the ratio of small and large cementite particles varied with the variation of heat treatment and working processes, resulting in a significantly improved strength of the studied high-carbon steel. The major conclusions were as follows:

(1) The microstructural features of the hot rolled 1.0C-1.5Cr steel mainly exhibited as netlike pearlite and pre-eutectoid carbides. The pre-eutectoid carbide mainly located at LAGBs. Close TEM observations showed that a large number of cementite particles with tens of nanometres in spherical interspersed among the lamellar cementite, and the thicknesses of lamellae varied from 10 nm to 20 nm.

(2) The morphology and size of cementite particles varied with different processes, especially the size distribution. After spherodizing annealing, the partially undissolved particles exhibited as elongated and irregular shapes because of the incomplete dissolution and unevenly distribution of cementite. The average size of cementite was the smallest at 450 nm, with the smallest ratio of large particles at 12%. In contrast, the average sizes of cementite after cold rolling and cold drawing were large at 630 nm and 600 nm because of the elongation along the rolling/drawing direction. The ratio of large particles was 25% and 28% after cold rolling and cold drawing, respectively.

(3) EBSD study showed that the frequency of LAGBs after cold drawing significantly increased to ~90%. The presence of these LAGBs was beneficial to the diffusion of elements, such as carbon and chromium, accelerating the coarsening of cementite. The dissolution and coarsening of cementite particles occurred simultaneously during cold working. The decreasing interface energy actuated the coarsening of cementite particles, while the LAGBs and high density of dislocations played a key role in the element diffusion.

(4) Cold deformation process affected the morphology and size of cementite particles, leading to different mechanical properties of the fine-grained ferritic steel. The steel containing spherical cementite showed a larger elongation compared to that containing lamellar cementite. After cold drawing, both *σ_y_* and *σ_UTS_* of the high-carbon steel (1.0C-1.5Cr-0.31Mn-0.20Si, wt %) were significantly high at 670 MPa and 740 MPa, respectively, accompanied by a good ductility of 26%.

(5) Finite element simulation showed that, at the beginning of each pass during cold rolling, the stress and strain increased with increasing depth from the surface to a depth of ~3 mm, and subsequently, the values decreased with the increased depth. The largest stress and strain were 830 MPa and 0.6 at a depth of 3 mm after the fourth pass of the 0.10C-1.50Cr steel, respectively. Stress and strain accumulation in the surface region of the cold rolled steel led to the existence of dense LAGBs.

## Figures and Tables

**Table 1:** Chemical composition of 1.0C-1.5Cr steel (wt %).

C	Cr	Mn	Si	S	P	Al	Ti	Fe
0.10	1.50	0.31	0.20	0.01	0.011	0.005	0.0023	Bal.

**Table 2:** Yield strength, tensile strength, elongation, and hardness of 1.0C-1.5Cr steel after various processes.

Process	Yield Strength ± 10 MPa	Tensile Strength ± 15 MPa	Elongation ± 1%	Hardness ± 0.03 GPa
Spherodizing annealing	340	640	48	1.42
Cold rolling	565	680	37	2.05
Recrystallization annealing	470	660	42	1.87
Cold drawing	670	740	26	2.36
Stress relief annealing	600	740	28	2.18

## Data Availability

The raw/processed data required to reproduce these findings cannot be shared at this time as the data also forms part of an ongoing study.
